# Negotiating Mental Health During the COVID-19 Pandemic: Performing Migrant Domestic Work in Contentious Conditions

**DOI:** 10.1177/00027642211000394

**Published:** 2021-09

**Authors:** Satveer Kaur-Gill, Yeo Qin-Liang, Samira Hassan

**Affiliations:** 1 National University of Singapore, Singapore

**Keywords:** (im)mobile migrants, labor precarity, culture-centered approach, mental health, migrant domestic workers, COVID-19 pandemic

## Abstract

Migrant domestic work is performed in precariously (im)mobile working conditions that mark the subaltern body in a state of constant lived experience with and in strife. In Singapore, the structural context of hire amplifies conditions of servitude, indebtedness, and subalternity that have implications for mental health. This study documents mental health narratives by migrant domestic workers during the COVID-19 pandemic, registering how mental health is negotiated amid dissension in the performance of precarious labor. While functional employment structures enabled and empowered well-being, dysfunctional structures disrupted mental health meanings, creating layers of constant contention for domestic workers to broker, limiting opportunities for mental health and well-being. Narratives gathered indicate systemic mental health precarities tied to workplace dysfunctions.

It’s not very easy to find a family that can fit me. Because for me I don’t want to rush anymore to find employers like I did. While I am looking for employer, I am looking at their background because I already got trauma. I rushed into this second employer. . . . You need to choose, because I don’t want to rush because if I find family I don’t know what will happen to me. I need to choose, that’s why I never find quickly. I don’t want to rush anymore.

Lyn is a domestic worker from the Philippines. In describing her mental health in the context of her employment conditions, Lyn reveals the layers of trauma-inducing mental health violence she experienced. The COVID-19 outbreak further amplified occupational hazards for migrant domestic workers (MDWs) such as Lyn because of the lockdowns. During the Circuit Breaker, Singapore’s version of the global lockdown during the COVID-19 outbreak, the cycles of mental health violence intensified for Lyn causing her to flee her employer’s home. Despite residing in a shelter during the interview, she reveals the importance of locating safe spaces of work as an MDW in Singapore. In the above quote, Lyn articulates the interplay of her mental health narrative as an MDW and the conditions of performing caregiving labor. During the Circuit Breaker Phase 1, MDWs were informed by the Ministry of Manpower (MOM) Singapore that they are required to stay at home on their off days (MOM, 2020a). In Phase 2, as eases on lockdowns took place, MDWs continued to be encouraged to stay at home on their off days, with employers having the capacity to determine domestic workers’ movement (MOM, 2020b; Paul, 2020). Amid already restrictive employment conditions, domestic workers now grappled with lockdowns that entailed taking a rest day within their employment context.

During the COVID-19 pandemic, the storied descriptions of mental health meanings among MDWs in Singapore highlight the role precarity amid (im)mobility in detailing mental health contentions. Occupationally related structures centered how MDWs discussed health descriptors in their role as caregivers during the interviews. This piece details the communicative registers of mental health precarity by domestic workers in conducting caregiving in the (im)mobile migration circuits of low-wage transnational labor (Dutta & Kaur-Gill, 2018).

## Migration and Domestic Work in Singapore

Scholars have long highlighted the role of precarity in the movement of MDWs from Southeast and South Asia to urban city centers such as Dubai, Hong Kong, and Singapore. Silvey and Parreñas (2019) conceptualize these fraught journeys as precarity chains with limited upward social mobility. Precarity chains, therefore, refer to such migrant jobs as financially insecure migration journeys that keep migrants in bonded debt cycles. These debt cycles, coupled with low-wage schemes, can leave migrants and their families in financial debt despite transnational mobility. According to Silvey and Parreñas (2019), “precarity chains effectively remit persistent dependence and future precarity on the families and household economies of these low-wage domestic workers, tending overall to reproduce the relative poverty, persistent socio-spatial precarity, and transnational subordination of domestic workers over the life-course” (p. 3458). Theoretically analogous, Dutta and Kaur-Gill (2018) apply the concept of (im)mobilities concerning the migration of low-wage migrant workers in Singapore. Here, the authors argue that the structural conditions of labor keep workers (im)mobile despite the acts of migration and mobility. These journeys are “ . . . determined by the systematic inhibitions placed on workers through exploitative employment practices . . . ” (p. 4077). Scholars have long documented the precarity in these systems of hire in Singapore (Koh et al., 2017; Silvey & Parreñas, 2019; Yeoh & Huang, 1998), studying how current policies leave workers without safeguards and vulnerable to marginalizing contexts. Dutta (2020) contextualizes such low-wage migrant journeys as precarities operating within a transnational political economy of extreme neoliberalism. Extreme neoliberalism, explained by Dutta (2020) references the hire of low-wage migrants by anchoring in maximum, free market ideologies applied in an authoritarian sociopolitical landscape. Extreme neoliberalism, therefore, disables opportunities for collectivization and unionizing of migrant workers facing jarring disparities by nature of the sociopolitical economy that hires migrant labor in repressive regimes. According to Dutta (2020), extreme neoliberalism in authoritarian context operates through “communicative inversions” (Dutta, 2014) that strategically erases communicative narratives of the margins by propping up symbolic gestures of materiality that obfuscates lived experiences of subaltern sites. Dutta (2020) summarizes,

as both a role model and pedagogue of extreme neoliberalism since the 1980s, Singapore has continually experimented with and perfected the techniques of authoritarian repression, exploitation of labor, and accumulation of primitive capital. It has invented the statecraft of disciplining labor and silencing dissent as model governmentality, while turning itself into the Asian gateway for transnational capital (p. 2).

Without spaces for organizing change, workers in disenfranchising employment contracts negotiate multifaceted precarities across various structural impediments. During the COVID-19 outbreak for example, foreign construction workers were afflicted by the infections at much higher rate than the local population (Shanker et al., 2020), resulting in serious challenges in the provision of healthcare for these workers. Thus, in negotiating health disparities, low-wage migrant workers reveal various structural gaps in seeking access, resources, and aid in crisis while performing labor as low-wage migrants in Singapore. The reproduction of deep inequalities faced by low-wage migrants is thus, also communicatively performed.

### Precarious Workers and Migrant Health

Hargreaves et al. (2019) meta-analysis identify the role of hazardous and exploitative labor conditions for international migrant workers worldwide, causing substantial health deprivation and illness. When migrants are employed in a political economy that accelerates and prioritizes labor productivity, health outcomes remain deleterious (Mao & Ahmed, 2018). In the context of Singapore and low-wage migrant workers, Kaur-Gill and Dutta (2020b) highlight the communicative tropes of productivity in the context of neoliberal governmentality that stretches the bodies of migrant workers as sites of extraction. In extracting labor, the health context of migrant workers in Singapore are imbued in “use and discard” policies of hire (Ong & Yeoh, 2013), where profit extraction is often central to the health vulnerabilities faced by migrant workers. Overworked by employers, limited rest hours, intermittent food insecurity, and violence are revealed as insights when detailing health vulnerabilities experienced by low-wage migrants as occupational lived experiences (Kaur-Gill & Dutta, 2020a).

A report by a local NGO in 2015 shared that 24% of domestic workers suffer poor mental health (Humanitarian Organization for Migration Economics, 2015), indicating mental health as a health disparity faced by MDWs in Singapore. Wong et al.’s (2020) experimental study on a mental health training program with Filipina domestic workers in Singapore revealed improvements with mental health when culturally specific mental health interventions addressing workplace inhabited the disparities. While Wong et al.’s (2020) study remains a critical intervention for migrant workers, they remain marginal in terms of reach as MDWs continue to remain (im)mobility erased from mainstream health resources and interventions.

Kaur-Gill and Dutta (2020c) qualitative insight on mental health meanings among Singapore’s MDWs detailed the nature of conducting migrant domestic work that renders migrant workers in a cycle of mental health trauma and suffering, attributable to occupational precarity. Therefore, in conceptualizing the mental health needs of MDWs, the occupational are read from the structures of precarity that shape mental health disparities (Kaur-Gill & Dutta, 2020c; Parreñas et al., 2020; Silvey & Parreñas, 2019; Wee et al., 2019). Thus, in theorizing health, the structural and cultural conditions of labor that domestic workers are employed within remain central in the analysis of mental health stressors discussed by domestic workers (Kaur-Gill & Dutta, 2020c).

Extensive literature unearths the multiple disparities faced by migrant workers across various cities around the globe, including Singapore (e.g., Anderson & Anderson, 2000; Parreñas, 2015; Wee et al., 2019). The health implications of performing low-wage migrant work are located in precarious labor conditions. For example, Ang et al’s. (2017) study of migrant construction workers in Singapore identifies the relationship between psychological distress to the nature of labor (high indebtedness owed to agents and financial insecurity). Views from migrant workers and doctors in Singapore reveal perceived barriers in how healthcare was delivered to migrant workers (Ang et al., 2017; Ang et al., 2019). Harrigan et al. (2017) study informed the role of migrant status in accessing predictors of mental health distress. The study surveyed and interviewed migrant workers in Singapore, revealing the role of deportation threats by employers when workers were in a workplace conflict. This was a critical predictor of risk to mental health. Therefore, central in the analysis is the role of occupational hazards in how migrant workers articulate mental health disparities.

During the COVID-19 pandemic, Chan and Kuan (2020) documented the unmet need for holistic care as a grave health threat, including mental health support for migrant workers in Singapore. Cultural and language barriers surfaced in meeting these needs. Thus, health responsiveness meant activating a variety of stakeholders to amass critical health care infrastructures that were tailored for migrant workers. Dutta’s (2020) study highlighted the jarring health disparities faced by migrant construction workers in meeting basic health care needs during the pandemic, such as sanitation, social distancing, and mental health care. With COVID-19 infections surging disproportionately among migrant construction workers in Singapore, various studies discuss the lack of social protection and occupational mismanagement as indicators of surging infections (Goh et al., 2020; Yi et al., 2020). Goh et al.’s (2020) study articulated the role of health disparities for migrant workers, including health information and language barriers as challenges faced in meeting the healthcare needs of these groups of workers that remain in the peripheries of Singapore society. Rajaraman et al. (2020) iterate the various social and structural factors that create substantial barriers for low-wage migrant workers in Singapore to access health care services, including costs, deportation threats by employers, and employers acting as gatekeepers in preventing workers from pursuing health care services. The study in detailing NGO accounts of migrant worker barriers to health care highlighted health risks to migrant workers as the potential collusion between health care providers and employers in medical leave provision to the worker during a health episode.

Lai and Fong’s (2020) randomized sample of *n* = 2017 MDWs in Hong Kong highlighted the relationship between the working conditions of domestic workers and abusive outcomes. Implications of performing caregiving within abusive settings compromise well-being among these groups of workers. Dutta et al. (2018) study of health meanings of domestic workers in Singapore concludes that structural constraints shape the agentic foregrounding of health choices. Therefore, migrant health is constituted amid rigid negotiations of cultural environments, structural limitations, and agentic performance and rituals of health. To break down the barriers in health care facilitation for low-wage migrant workers, a structurally responsive health model is signaled as central in domestic worker health meanings (Dutta et al., 2018; Kaur-Gill & Dutta, 2020c).

### Culture-Centered Approach to Migrant Health

Francisco’s (2014) participatory action research with MDWs highlighted the powerful role community-centered research plays in locating voice of migrant workers that are socially and politically attuned to their lived experiences. The culture-centered approach (CCA) rooted in community activated philosophies of knowledge production, considers solutions for health in subaltern sectors by locating methodological opportunities for dialogue. These methods include participatory action research, community-level in-depth interviews, focus groups and, advisory boards to create openings for listening to communities that are typically erased from mainstream theorizing of health (Dutta, 2014). Without communicative capacity, Dutta (2014) positions that marginalized spaces as absent from “mainstream communication theorizing and research” (p. 67). To bring back the subaltern in the solutioning of health interventions, their voices must be centered when making sense of health meanings contextually. By contextually placing these health meanings in conversation with tailoring better immigrant health strategies, the structure, culture, and agency nexus forms the frame of theorizing migrant health. Therefore, the fundamental crux of the CCA is the cyclical reading of culture, structure, and agency.

Culture-centered research on migrant health has yield insights on the ways occupational precarity informs health disparities. Gao et al. (2016) discussed how Chinese immigrants that partake in restaurant work in the United States indicate the role of work concerning health. The lack of knowledge on healthcare systems in the host country, such as health insurance schemes, limited their ability to access medical systems. Kumar and Jamil’s (2020) interrogation of health and labor of Bangladeshi migrant construction workers in the Middle East reveal how the labor economy fundamentally exposes class and race disparities that contribute to poor health opportunities. Kumar and Jamil (2020) summarize,

the pressures of migration from low-to high-income countries, including restrictive visa policies, and exploitative middlemen working at the behest of UAE employers, exacerbated by a blatant lack of labor protections once they arrive at their labor camps or sponsored employment location. (p. 1734)

Cao and Wang (2019) study of female migrant workers in China informs us how structurally articulated gaps such as a lack of a minimum wage and conditions of low-incomeness impede the ability of migrant workers to access essential health care services. Kumar’s (2020) study of Burmese refugees in the United States articulates how language isolation exacerbates health information disparities.

Dutta et al.’s (2018) report on health meanings of domestic workers in Singapore expand on how the invisibility of domestic work (located in the private sphere) amplifies health threats. Furthermore, the sociopolitical structures relating to the organizing of migrant domestic work create ripe opportunities for health deprivation by employers and agents hiring domestic workers in Singapore. Thus, narratives of health of domestic workers are often tied to abusive and exploitative labor conditions. Kaur-Gill and Dutta’s (2020c) study on culture-centered mental health meanings of MDWs identify how labor and employment structures are anchored in the descriptors of mental health narratives by workers. Disenfranchised workers narrativize that the absence of a rights framework exacerbates the violence in everyday care work (Dutta et al., 2014). Without the ability to navigate out of abusive employment conditions, workers discussed the amplification of mental health stressors. The structural abuses that limit the agentic negotiations of the worker, such as threats, confinement practices, and abuse, create a climate of fear and stress for workers. With several culture-centered studies on migrant health unpacking the various dimensions of structure in informing health inequalities, this research seeks to answer two key research questions in the time of the COVID-19 pandemic:

**Research Question 1:** How did MDWs negotiate caregiving work during Singapore’s circuit break (lockdown)?
**Research Question 2:** What were workers’ mental health meanings when navigating disrupted employment conditions during the pandemic?

#### Collecting Pandemic Narratives

The COVID-19 pandemic posed unique and critical challenges for conducting field research that is grounded in subaltern voice. As migrant workers across the globe faced precarious conditions amid lockdowns, the capturing of these narratives remained necessary. In Singapore, navigating and accessing MDWs amid the lockdown was a daunting task. MDWs were now confined to their employers’ homes and were not able to take the day off during this period. Confinement of domestic workers posed several complications for narratives elicited during fieldwork. Many might not have unsupervised access to use their mobile phones, some may have been unable to speak freely about their work conditions, and workers that did not have a private space in the home would have limited opportunities to share stressors.

We were able to navigate access points by adopting snowball and convenience sampling. Investigators conducted in-depth interviews with *n* = 32 female MDWs. After the lockdown was lifted, a nongovernmental organization (NGO; anonymized) also provided us with access points to domestic workers that were brought to the NGO shelter or domestic workers that ran away to the NGO to seek aid once the lockdown was lifted. Most in-depth interviews lasted between 30 and 90 minutes, depending on factors relating to work conditions. The demographic of the workers include 13 workers from the Philippines, 16 from Indonesia, and three from South Asia. All except two interviews (Punjabi) were conducted in English. We received approval from the National University of Singapore’s, institutional review board before commencing this research. All names have been changed to pseudonyms to protect our participants.

#### Data Analysis

We adopted Charmaz and Belgrave’s (2012) engagement with grounded theory in analyzing this dataset. The COVID-19 pandemic and global lockdowns remain a contemporary phenomenon that requires the development of new, in-depth, detailed, and contextual knowledge in theorizing migrant worker health. As the context of forced confinement centralized the lived experiences of domestic workers in Singapore during the Circuit Breaker, mental health meanings were analyzed in detail and without limiting conceptual possibilities. To pay close attention to the data in a way that was theoretically rich and meaningful, open, axial, and selective coding was employed (Charmaz, 2011).

Charmaz and Keller (2016) detail the organizing of data captured through open-ended; in-depth interviews can be done systematically through the process of coding. The coding process allows the adoption of a “heuristic device for engaging with the data and beginning to take them apart analytically” (p. 15). The open coding process read the data transcribed, line by line. The axial coding process shaped how mental health narratives during the lockdown were structurally configured. These included interpretations of open codes (e.g., scolding, salary, stress, worry, fear, touching, food), into broader categories (e.g., employment conditions, narratives of verbal abuse, docked wages, everyday threats, food insecurity, etc.). Employing the culture, structure, agency nexus at the stage of selective coding to theorize mental health disparities, themes on Benevolent Structures, Mental Health as Structural Precarities, and Employment Dysfunctions were theoretically anchored. The selective codes teased how the dialectics of structures manifested in the discourses on mental health narratives. This process led to a broad discussion on the contractions of Functional and Dysfunctional Structures of performing caregiving labor as a migrant with two subthemes on (a) Benevolent Structures and (b) Mental Health as Structural Precarities, (c) Structural Contentions as Lived Experience.

## Findings

### Performing Caregiving in Functional and Dysfunctional Structures

Our findings detail that the COVID-19 pandemic amplified the role of dysfunctional structures in how mental health meanings were discussed by domestic workers. In theme one, we see how domestic workers articulated different faces of the structure (various employment environments) regarding their mental health meanings during the Circuit Breaker phases. During these more restrictive phases, workers relived their experiences of working within confinement conditions as they had limited mobility outside of worksites. The performance of care work in the private sphere meant limited protections, amplified by stark power distance between the employer and employee relationships. Answering the research question on caregiving practices during restrictive lockdowns, MDWs were caregiving in environments that amplified vulnerabilities because of their limited mobility. Intersectionally, these vulnerabilities are heightened when domestic workers are female migrants from the Global South navigating contexts where the disparities of power are magnified. Narratives, therefore, reveal the relationship between mental health inequalities in the context of functional and dysfunctional structures experienced by various structural actors (agents, state actors, employers).

### Benevolent Structures

“I respect them and they respect me” (Rose, domestic worker, Philipines). In making sense of the narratives of mental health among various domestic workers, respect and benevolence by employers were referenced as central protagonists in their meanings of mental health, where the employment context shaped how discourses on mental health were articulated. In situations where workers resided in ideal employment conditions, mental health meanings centered the actors’ experiences in their employment context during the Circuit Breaker. Rose discussed her relationship with her employer in discussing mental health and well-being during the lockdown,

Oh, yes, ma’am! We never have the problem with regards to my day off and communicating with them, because my employer and I have a very “open” relationship like, I mean, I respect them, they respect me. Whatever I want, whatever they want, so our relationship is going smoothly and we’re “open.”

During the Circuit Breaker Phase 2 period, where the lockdowns were easing for the rest of the population, the MOM continued advising domestic workers to stay at home (MOM, 2020b). The discrepancy in the rule meant that domestic workers were not allowed to take their day off outside of their employment context, while the rest of the population could do so (Paul, 2020). Domestic workers live and work within their employer’s home, blurring multiple boundaries for domestic workers on employment schedule vis a vis rest hours. However, domestic workers like Joanne share that if one has a good employer, mental health is positively negotiated during the COVID-19 outbreak and the subsequent lockdown. She describes how her employer ensured her day off was met even during the Circuit Breaker period when she was unable to go out due to state policy,

. . . If I feel hungry, I just go to the kitchen and make my food and then when I saw the dishes there, I will wash that because since I’m eating also and I washing my plate also, so I will wash also their dishes but not too much, yeah. But my boss is nice. Actually my boss is very good. You know they are not like other employers that not very . . . the helper didn’t give them rest like that, so much work for them, but my boss is not like that actually. They are very nice.

Joanne shares that her employers do not allow her to wash their dishes on her off day during the Circuit Breaker. She attributes her mental health outcomes as positive because of her employment conditions that stipulate work days and rest days. She shares that when she is bored, her employers’ provide her with access to digital services for entertainment, “like more on seeing . . . holding the phone like watching movies like that, talk to your friends or more Facebook like that.” In situations where domestic workers were in fair employment contexts, and employers respected worker rights, mental well-being is prioritized.

Open and equitable communication between employer and employee, employee and family, and social support networks were a crucial factor in managing mental health stressors. Lyn reveals,

Because it’s more than a year I haven’t seen my family. So most of the time I feel homesick. I miss them. Everytime I have interview with employer, I ask them if they are open to me using phone after work. Because it is very important. You are working and sometimes you feel alone because you are not in your own country and are living with a different family. So after work, you just want to talk with your family to relieve your stress.

Access to social support resources such as the mobile phone enabled mental well-being of domestic workers. For May, social inclusion and participation by employers were centered in mental health meanings relating to well-being,

I feel supported [by employer]. They always brought me . . . I’m feeling I’m family here because my ma’am and sir, they care about me, about my family. Yeah, every one year, I go home two times, when my ma’am go to holiday they send me go home.

May shares that with social inclusion, employers were empathetic to the needs of their domestic workers. Equitable communication practices also meant greater opportunities for care communication between employers and employees, “my ma’am always want to know what happened with my family and always help my family, yeah . . . give my son present and help them and present my birthday.” These narratives of fair employment practices such as equitable communication practices, social support, and inclusion, as well as the participation of domestic worker voice in familial affairs resulted in empowering practices of mental health while caregiving.

### Mental Health as Structural Precarities

MDWs discussed a range of structurally tied stressors in conceptualizing mental health meanings. The Circuit Breaker during the COVID-19 outbreak revealed various structural factors that contributed to domestic workers’ mental health stressors. These stressors were codified as performing caregiving under extended work hours, restricted freedoms, verbal abuse, docked wages, disrupted social support networks, and stressors in locating opportunities to navigate out of poor employment conditions.

#### Migrant Indebtedness and Mental Health

Worried about their families back home, workers such as Nupur described living in a verbally abusive employment context and had to run away eventually. She describes her employment context,

I am always so scared after hearing employers words they use on me . . . everyday a lot of abuse (verbal) . . . I always so scared when they are scolding me . . . I finish my work also . . . not satisfied. . .keep scolding.

Nupur had taken a 3.5-month agency fee locally and 30 000 Taka in Bangladesh to come to Singapore for domestic work. As a single mother, migrating for domestic work remains a vital source of income for her son and sister’s family back home. She highlights that she needs to provide for her son’s education and cannot afford to face income loss. She felt trapped and sustained the abuse because of the money she needed. Her son lives with her sister, as both her parents have passed on. The inability to provide money for her son back home after she ran away from the abusive employment conditions had taken a further toll on her mental health stress. She reveals,

my sister is upset with me . . . I don’t have job now . . . so I can’t send 13 000 Taka back. My sister also no money because her husband only driver and also now lost his job . . . I so very worried . . . thinking about this.

Unable to find another job, Nupur remains at the shelter with limited success at finding a new employer. She stipulates that she will be repatriated back because of her educational qualifications, losing all the debt she paid to come to Singapore.

#### Abusive Cycles and Mental Health

Pushpa ran away from her verbally abusive employer the moment the Circuit Breaker was eased. Pushpa shares that despite running away, she still ended up in a cycle of abusive conditions. When she ran way, she was placed in an interim facility. She ended up describing living in that facility, “a*si bahot dardey didi* [we live in extreme fear sister]” in that place. Having faced constant verbal abuse at her employer’s home and only afforded sleep less than 4 to 5 hours a day, Pushpa ran away from the household to remove herself from the abusive conditions. Instead, she found herself in greater precarity at the interim shelter,

Nobody will want to come here if we are not protected. I ran from verbal abuse from my employer’s home, only to see FDWs like me facing sexual harassment in places we are supposed to feel safe . . . we were subjected to the caretaker sexually harassing us, talking about our private parts in lewd ways . . . keeping us in fear. He not letting us out of our rooms. He make fun of us when we requested pads or other female hygiene needs.

Pushpa’s narrative is one example that reflects the tremendous stressors placed on MDWs when navigating out of poor employment conditions. The layers of precarity manifest in narrativizing a climate of living in constant fear and feeling trapped in a cycle of poor conditions. These stressors are intimately tied to the precarious migration pathways that leave workers without due recourse. Pushpa shared how the fear of getting into trouble for speaking up against her various abusers leaves her vulnerable and in a constant state of anxiety, “I want to report and I don’t want to be fearful but what if I don’t find another job? I want to stay and work. I don’t want to be sent back home.” As the abuse escalates daily, Pushpa recounts, “never mind if I have to go back, I will go back, but I don’t want other girls to face this.” The cycle of marginality and powerlessness amplifies stress tied to the occupational labels of arriving as domestic workers. Radha reveals,

I have a case pending against me now . . . I don’t want to speak up about him (referring to the caretaker). I have to just let him say these things about my breasts as I have no way out.

Similarly, Simi, another MDW, documents how her mental stressors are deeply rooted in the powerlessness she feels when navigating health and communicative resources from her employer. Simi fractured her foot while rushing to her employer’s call to bring his children downstairs. Despite the doctor citing that her injury would require surgery and that she would not be able to perform her daily employment chores, her employer still made her conduct them. Her injury got worse in the process, leaving her in extreme pain and agony. Her employer instead suggested that she was dramatizing her pain. When she could not take it any longer, she decided to get out of this context. However, without navigational capital such as the appropriate communicative and informational resources for support centers, she called the police for help. She was placed in an interim hostel. As she was scheduled for an upcoming surgery, her employer tried to repatriate her back. When he met her for a mandatory hospital appointment issued by the state, he chided loudly at her “do you think it is your grandfather’s money that I am going to pay for your surgery.” She cited remaining in extreme anxiety, not knowing if she will get the medical treatment required or she will be sent back without any compensation for her injury.

### Structural Contentions as Lived Experience

Domestic workers shared the deepening precarity of employment conditions without the ability to take the day off outside of the employers’ home during the Circuit Breaker. Liana shares,

It is really a big change because they are all at home. I can’t even have an hour rest daily. So it’s like everyday start in the morning, looking after kids, cannot even get a full rest. Especially one hour rest daily. My only rest is to eat lunch and sometimes eat lunch around 3 p.m.

The extended work hours during the Circuit Breaker limited Liana’s ability to contact her family, “sometimes, I can’t contact them [family] anymore because I am too exhausted and I just want to rest. So I just go to bed and sleep.” Despite the extended work hours, Liana reported that her wages were also docked,

My employer ask me for favor to decrease my salary from $700 to $300 a month. They said they cannot go to work for now because need to stay at home and don’t have money to pay. Of course I didn’t agree, because I have one kid and family to feed. I told them that this is not right. But they insist to just give me something that they want to give. I don’t have any choice but to receive this money and send it to my family. Because I need to send something to them.

Liana reveals how her mental health is far more pronounced in poor conditions of employment. After leaving her employment context for the salary deductions and verbal abuse and threats, her mental health had further deteriorated. She explains,

Right now it’s different. Before I can send money to them. Now they send money to me. . . . Because I don’t have job and some of my savings in my bank account here are loosing because I use for my allowance. Sometimes I need to buy something, especially eat, so I use some of them.

Without money, she cannot agentically negotiate her health and well-being, creating even greater stress conditions.

Restricted freedoms for domestic workers included narratives of confinement practices that were now sanctioned by state policy in the advisories that limited movement outside of the employment context on their day off. Sulasi shares,

Right now if can, I just want to go out for a walk outside. In the condo area, just find some fresh air. . . . But still cannot it. You know, it’s not easy for us. I don’t want to keep asking because employer wouldn’t like. I tried asking before to go down for 10 mins, longest 30mins, to walk around. . . . But the longest I can go down is 10mins just to collect things then must go up. Because my friends ask whether want to go around jogging, but I cannot.

The day off policy of MDWs during the Circuit Breaker period disallowed MDWs from pursuing leisure activities within employment settings. In the later phases of the Circuit Breaker, family members from the same household were allowed to exercise together. Domestic workers by nature of their employment and a lack of family network locally were not able to partake in such activities, much less so with other domestic workers within their estate. Other domestic workers in the estate can often be critical sources of social support.

Narratives of verbal abuse among disenfranchised domestic workers we interviewed revealed how such abuses amplified mental stressors. As there was little room to navigate out of these conditions, workers like Jolyn shared,

They say to me “a lot of people out there dying and no work, but you just want to go home.” They always blaming the thing on me, “you have a salary, you are lucky that you are here working for us,” they say like that. I tell myself the pandemic has nothing to do with me. I also working hard with them, then they say like that.

In this narrative, Jolyn narrates how her employers repeatedly suggested that she should remain grateful for the job she has. For Jolyn, the lived experience with verbal abuse was contributing to her mental health decline. She details,

[during this pandemic] they have a lot of things to do and they have problems in their business, then they like to scold me and blame me for the virus. . . . Like they say I’m ungrateful, a lot of people homeless, already bankrupt and me I just want to go home.

She eventually decided to run away from the employment conditions as soon as she was able to. For workers like Jolyn, the very process of getting out of poor employment conditions during the Circuit Breaker phases were deeply connected to how they discuss stressors. Jolyn shares that she called the police to aid her leave,

I tell the police that I don’t feel safe in my employers’ house because they always say insulting language to me. The police came and they want me to call employer and agency, and put me in my agency for accommodation but the agency say no accommodation for me. They don’t have space.

She describes being extremely frightened till the police arrived,

When my employer is at the house, she’s knocking at my door very angry. Want me to come out of the room. And I’m so scared. And then the police came to assist me. I didn’t come out until the police come.

Part of navigating out of poor employment conditions included dealing with multilayered stressors. These stressors described included finding sufficient resources to navigate running away, such as who to call, where to run to, and what to take along with you when running away amid lockdown restrictions. All of which tie back to the lack of navigational capital, contributing to stress amplification. In the process of running away, workers describe fearing how their employers’ would respond when they navigate out of such employment conditions. Jolyn shares,

But you know, when I runaway because I took all my belongings with me, they say to the police that I took some items from them. Then the police invited me for questioning and took all my luggage and they check. But nothing ah. Then the police ask me what I want to add to my statement. I say “I never take anything from them. I just want to go home.”

Jolyn reveals that being powerless in this context includes being intentionally accused of wrongdoing with structural actors often suspicious of the domestic worker instead of employers,

Of course, they will take the side of the Singaporeans still. Because the [anonymized shelter] wrote down the things about [employer] taking out $200 and $60 top-up load from my monthly salary in my statement. . . . And then the [anonymized institution] says that these unnecessary things I don’t need to tell shelter. I say, “it’s okay I paid for it, shelter staff just writing down for me.” Still [anonymized institution] say no need these unnecessary things, so no more questioning, take less time. They still taking side of the employer.

Similarly iterated by Pushpa, “some good caseworkers take our side, they understand our problem . . . but some poor girls get caseworker that take employer side and never listen to our story . . . just say okay okay okay”. These interactions reinforce powerlessness and fear, causing workers to be trapped in a cycle of stress, worry, anxiety as structural actors constantly surveil them as actors for surveillance and interrogation. However, good caseworkers reflected narratives of empowerment and mental well-being, “he told us don’t worry, I will help you, you must not worry, we will take care of you.”

Disrupted social support was also shared by multiple domestic workers when discussing mental health in performing conditions of domestic work during the Circuit Breaker phases. Ena shares how her inability to speak with her family back home due to disruptions in movement amplified her stress and worry. Ena relates,

. . . in the beginning all the shops are close, so I cannot contact my family in the night because that time they don’t have WiFi, cannot go to the shop to buy mobile data because everything is close. I think almost a month, I call them by normal call, not by WhatsApp or video call, because everything is close. So it was really tough for me during the first month, because it affect me, I cannot call them . . . It’s just like normal call, but it’s very expensive. So I can only call 5 to 10 minutes like that.

Lyn too discusses the challenges of limited social support during the Circuit Breaker where they were unable to see friends,

We can chat over the phone, but we can’t see each other. . . . During normal days we can hang out together. But during this pandemic we cannot do that anymore. We don’t have the same off-days because some employers don’t want their maids to go off-day on Sunday because it is every crowded. They only go out weekday. And some go out on Saturday. So we don’t have the same days to go out. So we don’t see each other. . . . Unlike before during Sundays we go to church, go to Lucky Plaza.

Similarly, Ryze details how the initial phases of the Circuit Breaker affected social isolation,

The first month [of confinement] was quite hard because I cannot meet my friends and my employers also tell me not to talk to my friends. Then okay lah, I try my best not to talk to them. At least one meter apart. I always tell my neighbor to wear her mask and not talk to me too near, because my employer is watching me. They don’t like that I talk to you. That’s the rule ah. And then sometimes got police patrol. I also scared that they catch us. Summon $300 I *jialat [singlish expression]*.

The fear of navigating social support in physical spaces worried her, especially with the strict laws on movement in the initial phases coupled with employer surveillance. During this period, any form of physical conversation outside of a household for both locals and domestic workers could result in fines. Ryze, therefore, refers to these fines as the $300 in her excerpt. With limited movement and confinement during the Circuit Breaker, domestic workers’ mental health was referenced within the context of how working conditions manifested in greater surveillance and monitoring of workers, including increased workloads.

## Discussion

The findings in our article reveal the interplays of mental health meanings situated within a structural context of employment and a cultural environment that manifests unequal power relationships and indebtedness. During the Circuit Breaker, domestic workers in already poor employment conditions found themselves at greater precarity and limited agency. Furthermore, the analysis captures the temporalities of mental health meanings articulated within the performance of conducting migrant domestic work in confined working conditions. Precarious migrant journeys include vicious debt cycles, unethical agents, and corrupt employment practices, detailing the scripts of mental health stressors. Migrant workers share the stark mental health disparities faced when residing in dysfunctional structures. MDWs are hired within a political labor economy that perpetuates the cultural context of enormous power disparities between employers and employees, heightening abusive conditions (Huang & Yeoh, 2007; Lan, 2003). The very construction of their narratives of migratory indebtedness, powerlessness as everyday experience, and fear as incapacity attends to how mental health precarities are embedded in functioning of labor (im)mobilities rooted in a state of constant contention.

These conditions of contentionality are anchored in the structural precarity of conducting domestic work. Migratory indebtedness is imbricated and fashioned for dependency (Lazzarato, 2015) that normalizes fear and powerlessness. Thus, mental health precarities must be theorized from the perspective of migrant “precarity chains” (Silvey & Parreñas, 2019) and (im)mobilities (Dutta & Kaur-Gill, 2018) that constructs and circulates fear as disciplining strategies. Fear and stress in performing migrant domestic work are rooted in a political economy that produces and circulates contentious labor conditions. Hence, mental health suffering as health violence specifically manifests in occupational conditions of contentions. Workplace contentions operate through dysfunctional structures that have limited safeguards for domestic workers to secure mental well-being. Thus, narratives by domestic workers on mental health meanings are constructed in the storied descriptions of trauma, pain, everyday memories of violence in the constitution of mental health suffering. Performing domestic work is, therefore, a performance of mental health in constant contention. In navigating dysfunctional structures, migrant domestic workers are in a constant state of assault and confrontation from multiple structural actors and employers. The navigating in and out of disruptive and abusive employment contexts point to domestic workers negotiating communicative contentions that have disrupted mental health outcomes for migrant workers.

For migrant workers who articulated good employment contexts, communicative equitability and respect remained central in the narratives of mental health well-being. Employers were either key protagonists or detailed as antiheroes that enabled or disabled empowering health opportunities. When structural actors were central protagonists in the storytelling of good mental health during the COVID-19 lockdown, workers were agentically empowered to secure necessary mental health resources. These workers had access to technologies and used technological devices to connect with critical social networks for support and were provided with technological access and use, for entertainment during nonworking hours and rest days.

Mental health suffering was especially prevalent in structural contexts that were disempowering, including violent and abusive employment conditions, culturally and structurally unresponsive caseworkers of MDWs, and shelter conditions that do not attend to vulnerabilities of low-wage migrant women. A culture, structure, agency analyses of mental health meanings thus, theoretically situates how workers constructed their experiences of mental health within the ambits of social, cultural, and structural contentions of employment as lived experiences in performing caregiving.

### Practical Implications

Parreñas et al. (2020) findings among Singaporean employers on their domestic workers reveal employers center maximum labor extraction that impede work friendly policies. Soft violence is a strategy employed to keep the practices of servitude in place. Our findings reveal that the COVID-19 pandemic lockdowns magnified how these practices of soft violence (Parreñas et al., 2020) manifested, disabling migrant worker mental health and well-being. As precarity is structured in the power imbalance to hire, fire, and deport domestic workers at will by employers, practical implications for mental health interventions must discuss labor and immigration policies of MDWs bound to Singapore. The culture of erasure, bonded labor via debt precarity, the blurring boundaries of work and rest hours, and occupationally hazardous environments (removal of mobile phone, social networks, abuse) are embedded in the migration regime of MDWs to Singapore. Without changes to these fundamental structural mechanisms of labor, mental health and well-being of domestic workers will center around occupational related health hazards. Beyond culturally tailored programs on mental well-being in the workplace, solutions must consider the structural factors embedded in the policies of hire and the indentured nature of labor to address MDWs discussions of mental health contentions.
